# A Structure-Based Allosteric Modulator Design Paradigm

**DOI:** 10.34133/hds.0094

**Published:** 2023-12-15

**Authors:** Mingyu Li, Xiaobin Lan, Xun Lu, Jian Zhang

**Affiliations:** ^1^ College of Pharmacy, Ningxia Medical University, Yinchuan, NingxiaHui Autonomous Region, China.; ^2^ State Key Laboratory of Medical Genomics, National Research Center for Translational Medicine at Shanghai, Ruijin Hospital, Shanghai Jiao Tong University School of Medicine, Shanghai, China.; ^3^ Medicinal Chemistry and Bioinformatics Center, Shanghai Jiao Tong University School of Medicine, Shanghai 200025, China.

## Abstract

**Importance:** Allosteric drugs bound to topologically distal allosteric sites hold a substantial promise in modulating therapeutic targets deemed undruggable at their orthosteric sites. Traditionally, allosteric modulator discovery has predominantly relied on serendipitous high-throughput screening. Nevertheless, the landscape has undergone a transformative shift due to recent advancements in our understanding of allosteric modulation mechanisms, coupled with a significant increase in the accessibility of allosteric structural data. These factors have extensively promoted the development of various computational methodologies, especially for machine-learning approaches, to guide the rational design of structure-based allosteric modulators. **Highlights:** We here presented a comprehensive structure-based allosteric modulator design paradigm encompassing 3 critical stages: drug target acquisition, allosteric binding site, and modulator discovery. The recent advances in computational methods in each stage are encapsulated. Furthermore, we delve into analyzing the successes and obstacles encountered in the rational design of allosteric modulators. **Conclusion:** The structure-based allosteric modulator design paradigm holds immense potential for the rational design of allosteric modulators. We hope that this review would heighten awareness of the use of structure-based computational methodologies in advancing the field of allosteric drug discovery.

## Introduction

Allostery, or allosterism, was first discovered by Bohr and colleagues over a century ago, prompted by their observation of positive cooperativity in the binding of oxygen to the subunits of the hemoglobin protein [1]. Subsequent decades of exploration have consolidated the notion that allostery is not merely confined to the cooperativity between protein subunits. Instead, it is an intrinsic attribute of all flexible biomolecules, mainly proteins, wherein orthosteric sites (functional sites) exhibit functional coupling over considerable distances with topologically disparate regions, known as allosteric sites. Stemming from the allosteric sites, the allosteric signal propagates to the orthosteric sites in a wave-like manner. This propagation is observed along the interaction pathway of residue networks, often referred to as the allosteric pathway, and is mediated by conformational dynamics within the protein. The overall process regulates the protein’s biological activity, a mechanism commonly known as allosteric regulation [2]. Allosteric regulation is instrumental in a plethora of processes, spanning from molecular-level activities such as enzyme catalysis to more comprehensive cellular events like proliferation, extending even further to the maintenance of systemic homeostasis [2,3].

In the realm of pharmaceutical science, allosteric regulation is emerging as a revolutionary model in the development of therapeutics, particularly for addressing seemingly intractable protein targets. Historically, many crucial protein targets have been considered undruggable or difficult to target via traditional orthosteric drug design [4,5]. This has primarily been due to obstacles such as intense competition with highly affinitive endogenous ligands like the Ras protein superfamily [6], the absence or malformation of orthosteric binding sites within protein–protein interaction (PPI) regions [7], and selectivity challenges posed by highly conserved orthosteric sites across protein superfamilies, such as G-protein-coupled receptors (GPCRs) and protein kinases (PKs). Allosteric therapeutics offer a viable alternative pathway to circumvent these hurdles, exhibiting several advantages over their orthosteric counterparts. Firstly, allosteric modulators can bypass stubborn competition in the orthosteric sites or flat PPI sites by binding to a distinct allosteric site. Secondly, as allosteric sites tend to be less conserved than orthosteric sites, allosteric modulators exhibit a high subtype selectivity and specificity, thereby minimizing off-target adverse effects [8,9]. Thirdly, the strategic combination of allosteric and orthosteric modulators could potentially overcome the obstacles presented by drug-resistant mutations [10,11]. In addition, as exemplified by GPCR-biased allosteric modulators, they can stabilize a specific protein conformation, facilitating bias signaling and precise therapeutics [12,13]. Finally, allosteric modulators operate non-competitively, allowing endogenous ligands to bind and cooperatively fine-tune protein activity, which results in topical, localized actions without requiring complete activation or deactivation [14].

The heightened pharmaceutical interest in this area has rapidly transitioned the discovery of allosteric modulators from serendipity to a more systematic, “rational” design approach [8,9,15]. This is executed through the combination of computational and experimental methodologies, particularly the structure-based allosteric drug design (SBADD). SBADD serves as a pivotal component of the initial stages of drug discovery, integrating 2 critical disciplines of allosteric drug discovery: structural biology, corresponding to the “structure-based” aspect, and bioinformatics, which is aligned with “design”. The fundamental first step in SBADD is the determination of the target protein. Excitingly, experimental crystallographic data—spanning x-ray, nuclear magnetic resonance, cryogenic electron microscopy, and more—have accumulated more than 100,000 unique protein structures [16]. Specifically for allosteric resources, the Allosteric Database (ASD) platform has comprehensively collected over 100,000 allosteric modulators and nearly 2,500 co-crystals [17–20]. Meanwhile, one of the representative breakthroughs in bioinformatics, Alphafold2 (AF2), has utilized powerful machine learning (ML) technologies to computationally produce experimental comparable protein 3-dimensional (3D) structures and has completed the human proteome structure predictions [21]. Hence, acquiring 3D structures of therapeutically important protein targets has become considerably accessible and convenient.

The subsequent step of SBADD is to identify potential allosteric binding sites. Prior to this, it is essential to survey existing literature to determine if an allosteric site for a given protein has already been described. The ASD serves as a central repository of relevant literature and annotations relating to allostery, offering a rich trove of information for this exploratory step. If no allosteric site has been previously identified, site detection algorithms can be readily used to detect visible putative allosteric cavities in the protein targets. However, allosteric sites are often hidden (cryptic) in the static crystals and emerge transiently within the protein conformational ensembles [22]. To discover such cryptic cavities, coarse-grained normal mode analysis (NMA) [23] and all-atom molecular dynamics (MD) simulations [24] are commonly used, enabling us to move beyond the static limitations of the crystals and generate dynamic conformational landscapes of the targets. An often-neglected factor during this phase is these newly identified allosteric putative cavities’ druggability—that is, whether these cavities can communicate with the orthosteric site or other functionally important regions, thereby regulating protein function upon ligand binding. Upon identifying regulatable allosteric sites, the next phase is to find their allosteric modulators. This can be achieved either by selecting candidate ligands from known libraries based on a binding assessment or by designing candidates de novo with desirable properties.

**Fig. 1.**
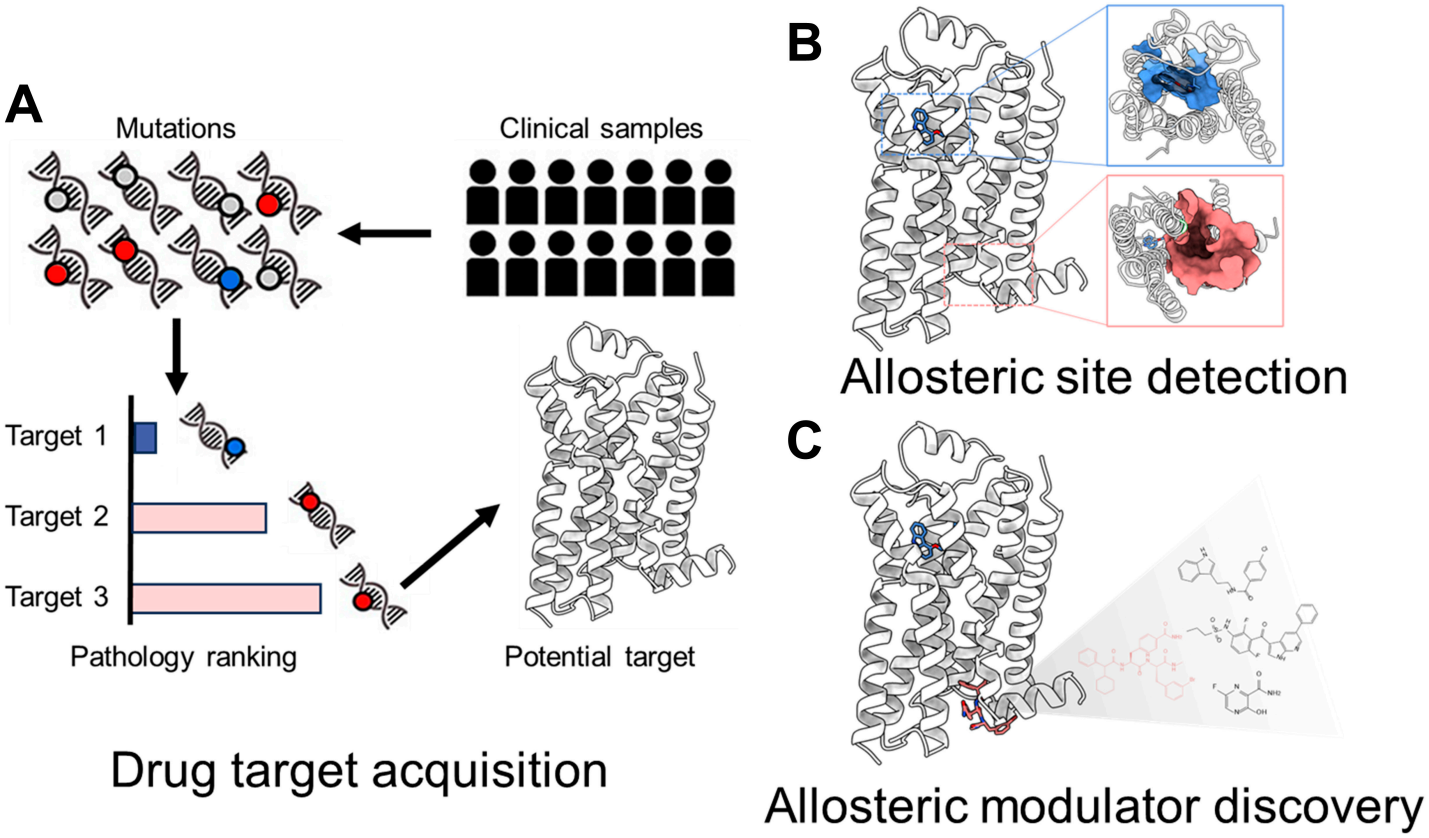
An overview of SBADD tasks in this review. (A) Drug target acquisition, the first step in SBADD, aims at identifying potential therapeutic target proteins from extensive clinical sequencing data and obtaining their 3D structures. (B) Allosteric site detection, the subsequent step, focuses on identifying druggable allosteric binding cavities within the target 3D structures. (C) Allosteric modulator discovery, the final phase, seeks to discover candidate allosteric modulators for the identified pockets through screening or de novo design.

In this review, we present an overall paradigm of SBADD along 3 primary stages: allosteric drug target acquisition, binding site identification, and modulator discovery (Fig. 1). For each stage, we conduct a systematic survey on recent computational methodology advancements (Table). Subsequently, 2 quintessential examples of SBADD are provided, employing the reviewed computational platforms in combination with experimental validation. Finally, we outline the challenges impeding the discovery of allosteric modulators and propose future perspectives to stimulate progress in the field of allosteric modulator discovery.

**Table.** Allosteric database and computational platforms for allosteric target acquisition, site identification, and modulator discovery

**Allosteric database**	**Website URL**	**Description**
ASD [17–20]	http://allostery.net/ASD/	Versatile resource for structure, function, disease, and related annotation for the allosteric macromolecules and allosteric modulators.
ASBench [42]	https://mdl.shsmu.edu.cn/asbench/	Benchmarking dataset for experimentally determined allosteric sites.
AlloMAPS [37,38]	https://allomaps.bii.a-star.edu.sg/	Versatile resource for the energetics of coupling, including allosteric regulation, allosteric signaling, and allosteric effects of mutation in proteins.
AlphaFold DB [100]	https://alphafold.ebi.ac.uk/	Versatile resource of over 200 million protein 3D structure predictions.
**Allosteric drug target acquisition**	**Website URL**	**Description**
AF2 [21]	https://github.com/deepmind/alphafold	Stand-alone tool predicting protein 3D structures.
RoseTTAFold [25]	https://github.com/RosettaCommons/RoseTTAFold	Stand-alone tool predicting protein 3D structures.
ESMFold [26]	https://github.com/facebookresearch/esm	Stand-alone tool predicting protein 3D structures.
AlloSigMA [34,35]	http://allosigma.bii.a-star.edu.sg/	Web-server tool utilizing SBSMMA to quantify energetics of allosteric signaling and mutation and to predict allosteric site by bidirectional allostery.
AlloDriver [39–41]	http://www.allostery.net/DeepAlloDriver/	Web-server tool utilizing ML to provide therapeutic targets from cancer samples.
**Allosteric binding site identification**	**Website URL**	**Description**
AlloSite [44]	https://mdl.shsmu.edu.cn/AST/	Web-server tool combining static features for ML model training in allosteric site prediction.
ALLO [46]	https://github.com/fibonaccirabbits/allo	Stand-alone tool combining static features for ML model training in allosteric site prediction.
PASSer [45]	https://passer.smu.edu/	Web-server tool combining static features for ML model training in allosteric site prediction.
AlloSitePro [47]	https://mdl.shsmu.edu.cn/AST/	Web-server tool combining dynamic features (NMA) for ML model training in allosteric site prediction.
AlloPred [48]	http://www.sbg.bio.ic.ac.uk/allopred/home/	Web-server tool combining dynamic features (NMA) for ML model training in allosteric site prediction.
NACEN [49]	http://sysbio.suda.edu.cn/NACEN/	Stand-alone tool combining topological features extracted from node-weighted residue interaction networks for ML model training in allosteric site prediction.
PARS [50]	http://bioinf.uab.cat/cgi-bin/pars-cgi/pars.pl	Web-server tool combining dynamic features (NMA) for allosteric site identification.
ESSA [52]	http://prody.csb.pitt.edu/essa/	Web-server tool combining dynamic features (NMA) for allosteric site identification.
STRESS [51]	https://github.com/gersteinlab/STRESS	Stand-alone tool combining conformational change sampled by Monte Carlo simulations and topological features for allosteric site identification.
P2Rank [53] and PrankWeb [54]	https://prankweb.cz/	Web-server and stand-alone tool identifying and clustering ligandable points into surface patches for predicting allosteric sites.
BiteNet [55]	https://sites.skoltech.ru/imolecule/tools/bitenet/	Web-server tool combining computer vision theory for ML model training in allosteric site prediction.
JEDI [59]	https://github.com/michellab/jedi-utilities	Stand-alone tool utilized collective variables dependent on enhanced sampling for cryptic allosteric site prediction.
SWISH [60]	https://github.com/OleinikovasV/CrypticSWISH	Stand-alone tool utilized collective variables independent on enhanced sampling for cryptic allosteric site prediction.
MDmix [61]	https://mdmix.sourceforge.net/	Stand-alone tool utilized mixed-solvent MD simulations method using probe molecules for cryptic allosteric site prediction.
CryptoSite [64]	https://modbase.compbio.ucsf.edu/cryptosite/	Web-server and stand-alone tool utilizing MD simulations and ML model for cryptic allosteric site prediction.
PocketMiner [65]	https://pocketminer.azurewebsites.net/	Web-server and stand-alone tool utilizing MD simulations and ML model for cryptic allosteric site prediction.
AlloReverse [70,71]	http://www.allostery.net/AlloReverse/	Web-server and stand-alone tool utilizing bidirectional allosteric theory, NMA, and ML model for cryptic allosteric site prediction.
CorrSite [72,101]	http://www.pkumdl.cn/cavityplus/	Web-server and stand-alone tool utilizing bidirectional allosteric theory and NMA for cryptic allosteric site prediction.
D3Pocket [73]	http://www.d3pharma.com/D3Pocket/	Web-server and stand-alone tool utilizing bidirectional allosteric theory to discover crosstalk between allosteric sites and orthosteric sites.
**Allosteric modulators discovery**	**Website URL**	**Description**
Alloscore [79]	https://mdl.shsmu.edu.cn/alloscore/	Web-server tool combining an ML model to predict binding affinities of allosteric protein–modulator interactions.
AlloType [80]	https://github.com/qj-Huang/AlloType	Stand-alone tool combining the forces of protein-modulator binding to predict allosteric type.
LiGANN [85]	https://playmolecule.com/LiGANN/	Stand-alone tool combining ML model to de novo design modulators.
AlloFinder [90]	https://mdl.shsmu.edu.cn/ALF/	An integrated allosteric modulator platform.

## Allosteric Drug Target Acquisition

### Target 3D structure obtainment

Securing high-quality target protein 3D structures underpins SBADD, but obtaining them through experiments can be costly and challenging. Instead, the boost of ML technologies, epitomized by AF2, has engendered monumental advancements in the accurate comparable prediction of structures, comparable to experimental standards [21]. Further advancements include RoseTTAFold, which is proposed as a “3-track” model to exchange information among 1D multiple sequences, 2D pairwise distances, and 3D protein coordinates for precise protein structure prediction [25]. ESMFold, another development, utilizes an evolutionary-scale language model to expedite and accurately predict structures from individual sequences [26]. These ML-based protein structure prediction models typically comprise 4 primary components: an input sequence loader, a sophisticated ML algorithm to process spatial and evolutionary patterns, an output structure predictor, and a refinement module for structural fine-tuning [27].

While these methodologies have revolutionized structural biology, caution is required when using structures predicted by AF2 for SBADD. AF2 often generates structure in its inactive state, containing poorly defined allosteric sites and inaccurate side-chain orientations [28]. However, the inaccurate pocket characterization from AF2 could be adjusted by MD simulations or induced-fit docking with a known ligand [29]. Moreover, recent studies reveal that the inactive-bias recognition pattern of AF2 can be altered. When fine-tuned on a templated GPCR database annotated with distinct states, AF2 can predict the inactive, active, or even intermediate conformations of GPCRs at the atomistic resolution, describing the common structural motif changes along GPCR activation. The results also demonstrated that these multi-state models could be more suitable for GPCR-ligand docking than the original AF2 models [30].

### Novel therapeutic target identification

Novel therapeutic targets can be discovered by associating allosteric residues with certain pathological conditions and revealing the underlying mechanisms, such as pathological single-nucleotide polymorphisms (SNPs), particularly with the availability of enormous clinical sequencing data [31]. While the consequences of SNPs in catalytic or binding sites are thoroughly elucidated, our comprehension of mutations at allosteric sites remains limited [32]. To quantitatively elucidate the allosteric signaling upon mutation, Guarnera and Berezovsky [33] first proposed a computational framework known as the structure-based statistical mechanical model of allostery (SBSMMA). This model permits the evaluation of the energetics of allosteric effects at the per-residue level upon perturbations, such as ligand binding and/or mutations. In particular, the protein configurations before and after perturbations are represented using 2 harmonic Cα models. Utilizing NMA, for each configuration, 2 sets of low-frequency normal modes are derived to calculate each residue’s allosteric potential. This quantifies the average impact on a residue resulting from changes in its adjacent residues, which had been influenced by allosteric disturbances. To facilitate the use of SBSMMA, they further built the AlloSigMA web server [34,35]. Using AlloSigMA, they performed a comprehensive residue-to-residue description of allosteric communication in 27 proteins with numerous pathological SNPs [36]. Many of these SNPs were found to exert significant allosteric modulation on the protein catalytic activities, ultimately leading to downstream pathologies. These pathology-related SNPs and their allosteric effects were integrated into the AlloMAPS database [37], which was recently updated to include structures from the AF2 database and Pfam domains predicted by trRosetta [38].

Another attempt to investigate the pathology-associated allosteric regulation is the AlloDriver, a platform for identifying and prioritizing allosteric driver mutations of cancer targets developed by our laboratory [39–41]. AlloDriver takes raw cancer sample data as input, extracts variants, maps them to 3D protein structures, employs a well-trained ML model to recognize intricate functional patterns directly from structure data, and finally evaluates potential allosteric driver variants. Importantly, it predicted multiple novel driver variants among unreported targets across a wide spectrum of tumors, including non-small cell lung carcinoma and head and neck squamous cell carcinoma, among others. These driver mutations were further experimentally validated, and their corresponding proteins could potentially serve as novel therapeutic targets for cancer. Collectively, these user-friendly platforms could not only reveal novel molecular mechanisms of disease progression but also assist in identifying novel pathological targets.

## Allosteric Binding Site Identification

### Novel putative allosteric site discovery

Allosteric sites are generally categorized into visible or cryptic sites, depending on their detectability within experimentally determined structures. Visible allosteric sites are well-defined in the protein crystal structures, which have been meticulously collected and organized into our ASD and ASBench [42] data platform. Such platforms have been extensively used for training and testing numerous computational site detection tools [43]. Most of these tools regard allosteric site prediction as a classification task. The geometry-based pocket detection methods, such as Fpocket, Cavity, and LIGSITE, are first applied to identify potential cavities. Next, these cavities are featured and labeled as allosteric or non-allosteric by an ML model. Traditionally, some of these tools (e.g., Allosite [44], PASSer [45], and ALLO [46]) only extract the static features, such as cavity conservation, cavity volume, and physicochemical descriptors, as input features for ML model training. Later, to improve the predictive ability of the ML model, dynamic features calculated by NMA-based perturbation (e.g., AlloSitePro [47] and AlloPred [48]) and topological features extracted from amino-acid contact networks (e.g., Node-weighted Amino acid Contact Energy Network [NACEN] [49]) are combined for better allosteric site pattern leaning. Also, some methods, such as Protein Allosteric and Regulatory Sites (PARS) [50], STRucturally identified ESSential residues (STRESS) [51], and Essential Site Scanning Analysis (ESSA) [52], analyze the dynamic features and topological features without adopting ML, proposing a statistical criterion for allosteric site identification. In addition, a distinct strategy includes representing the protein with a 3D grid, identifying likely ligandable points, and then clustering the surrounding points into surface patches as the predicted binding sites. Platforms like P2Rank [53] and PrankWeb [54] employ this strategy. Another innovative tool, BiteNet [55], treats the 3D protein conformation as 3D images, regarding allosteric site detection as an object detection exercise within these images.

**Fig. 2.**
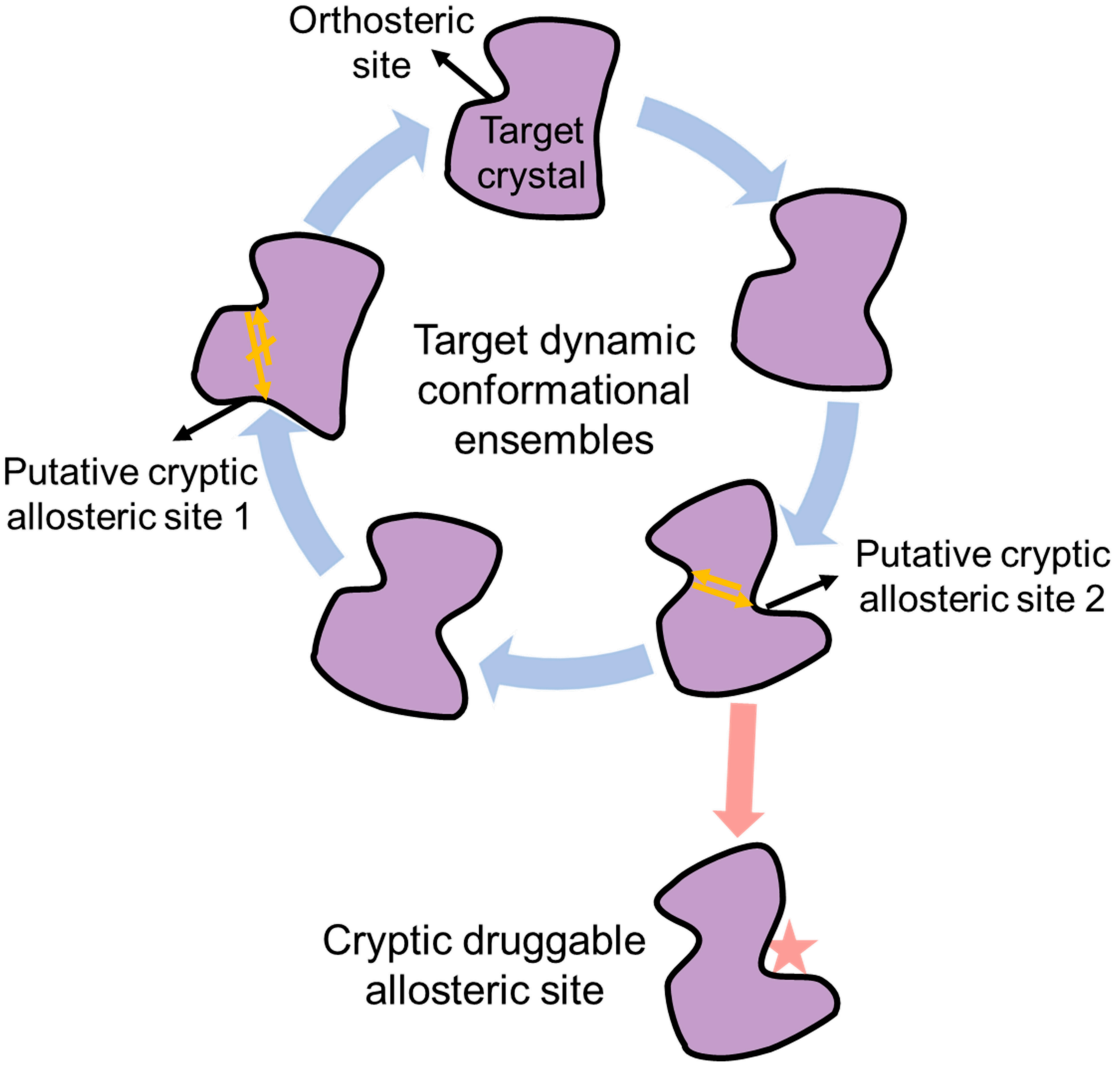
Illustration of the emergence of cryptic allosteric sites within the protein conformational ensembles. In the targeted crystal structure, no visible allosteric site is present. By exploring the conformational ensembles of the target protein, 2 putative cryptic allosteric sites appear. Only cryptic allosteric 2 is considered to be druggable owing to the existence of coupling (indicated by the orange bidirectional arrows) between this site and the orthosteric site.

Contrary to visible allosteric cavities, cryptic allosteric sites only transiently re-emerge with the protein’s latent conformational space [56]. To capture these cryptic sites, protein structural fluctuations are usually explored through MD simulations (Fig. 2) [57]. On the one hand, a recent study attempted to efficiently unveil the cryptic allosteric site by designing an allosteric protein-specific force field (APSF) [58]. In 8 representative systems, simulations based on APSF have successfully re-emerged known allosteric sites, including cryptic sites, which are challenging to identify under conventional force fields. On the other hand, applications of conventional MD simulations leverage enhanced sampling strategies (e.g., collective variables dependent as Just Exploring Druggability at protein Interfaces [JEDI] [59] or independent as Sampling Water Interfaces Through Scaled Hamiltonians [SWISH] [60]) for enhanced sampling the cryptic cavity opening events, mixed solvent (e.g., MDmix [61]) relying on small probes to facilitate and stabilize cryptic cavity formation, or Markov State Models disclosing protein conformational landscapes for recognizing the state with cryptic cavity appearance [62,63]. Moreover, recent advancements that combine MD sampling and ML algorithms for predicting cryptic pockets, such as CryptoSite [64] and PocketMiner [65], offer more rapid and cost-effective platforms. Notably, the application of PocketMiner to the human proteome revealed that over half of the proteins lacking visible cavities are likely to harbor cryptic ones, underscoring the vast potential of cryptic cavities in propelling allosteric drug discovery efforts.

### Evaluation of allosteric site’s druggability via bidirectional allostery

A critical assessment after identifying the new cavities is their druggability. Druggable allosteric sites can communicate with orthosteric sites or functionally crucial regions, thereby acting to modulate protein activity upon modulator loading [66,67]. Also, the examination of reverse allosteric communication from the orthosteric sites can also potentially unveil new allosteric sites based on bidirectional theory. Such explorations have been pioneered by Berezovsky et al. with the SBSMMA framework. They assessed the effects of orthosteric perturbations, confirmed the reversibility of allosteric communications, and rediscovered known allosteric sites in 13 quintessential proteins. This framework’s validity was further validated using a diverse set of 41 allosteric proteins from ASBench [68]. Very recently, they employed this approach to investigate bidirectional communication in 2 major drug targets: GPCRs and PKs [69]. The investigation yielded several potential allosteric sites and fragments for subsequent allosteric modulator designs. AlloReverse is another state-of-the-art computational pipeline developed by our laboratory [70,71]. The unbiased MD simulations of systems with (*holo*) and without (*apo*) orthosteric ligand loading were first performed. Comparative free energy analysis of these 2 systems resulted in an energy coupling score, reflecting the changes in free energy and the strength of allosteric crosstalk within the protein complex triggered by orthosteric ligand binding. A cluster of neighboring residues exhibiting high scores is likely to represent potential allosteric sites [70]. Furthermore, we developed the bidirectional allosteric regulation theory and extended the scope of AlloReverse by combining protein dynamics and ML model, revealing hierarchical coupling among all sites within one protein [71] as well as identifying cryptic sites. Remarkably, AlloReverse has facilitated the discovery of a range of novel allosteric sites in diverse proteins, including Cell Division Cycle 42 GTP-binding protein, Sirtuin 3 (SIRT3), and Sirtuin 6 (SIRT6). The functionality of the predicted allosteric sites has been validated experimentally through site-directed mutagenesis [70,71]. Additional computational methodologies have been developed to illuminate the coupling between orthosteric and allosteric sites in terms of their structural movement characteristics (e.g., CorrSite, featured in the CavityPlus, an integrated platform for site detection [72]) or biophysical properties (e.g., D3Pocket [73]). Of note, Li et al. [74] applied CorrSite to identify a potential allosteric site in Glutathione peroxidase 4 (GPX4). Following this discovery, they proceeded to design a GPX4 activator, known as 1d4, via virtual screening and medicinal optimization. The binding positions of 1d4 were further confirmed through the mutation of surrounding residues. In cell-free assays, this activator remarkably increased GPX4 activity to 150% at 20 μM.

## Allosteric Binding Modulator Discovery

### Binding assessment of allosteric modulators

The discovery of allosteric modulators fundamentally depends on the capacity to identify common characteristics inherent to known allosteric ligands or specific allosteric binding patterns with the receptor. Regarding ligand modeling in the absence of receptor information, Xie et al. [75,76] scrutinized the combination of various ML models and features of small molecules for binary classification in a cannabinoid (CB) receptor aimed at differentiating allosteric ligands from orthosteric ligands. Following this, they implemented multinomial classification within a broader GPCR system, which encompassed 10 GPCR subtype allosteric modulators and a random-type compound. In a parallel endeavor, Miljkovic et al. [77] employed various ML algorithms to train models for distinguishing between type I, I1/2, II, and allosteric (III or IV) PK inhibitors. Their best ML models demonstrated commendable performance, suggesting their potential applicability in identifying GPCR and PK subtype allosteric modulators.

On the other hand, additional computational methodologies are available for quantitatively evaluating the binding affinity, types, and efficacy of allosteric modulators with their receptors. A particularly efficacious strategy for appraising the affinity of binding modulators involves utilizing empirical interaction terms, or scoring functions, forming the cornerstone of high-throughput virtual screening [78]. Alloscore is the first allosteric-specific scoring function, which leverages 6 elaborately selected energy terms [79]. It demonstrated superiority over 25 traditional scoring functions in an external test set, offering a robust platform for ranking and selecting potential hit compounds based on their binding affinity in practical applications. Very recently, Huang et al. [80] proposed that the binding types (activation or inhibition) of allosteric modulators could be ascertained by the forces exerted within the pocket upon ligand loading and designed AlloType. It utilized an anisotropic network model (ANM) for coarse-grained characterizing the protein structure’s response and linear response theory to gauge structural fluctuations upon ligand loading. The force distribution within pocket residues is subsequently evaluated to predict allosteric modulatory impacts. In 13 of 16 representative allosteric systems tested, the types of allosteric modulators were classified, thus validating their reliability. AlloType marks an initial step toward designing allosteric drugs with the desired modulatory impacts. Calculating binding efficacy is a considerably challenging task, heavily reliant on system dynamics or kinetic changes. Ferraro et al. [81] offered an MD–ML framework for elucidating allosteric ligand efficacy as a function of localized dynamic patterns. The initial step of this framework is to conduct comparative MD simulations of *apo* and *holo* (inhibitor-bound) systems to generate local dynamic features. These features are subsequently incorporated into ML models to differentiate between apo and holo-like (inhibitory-like) states. They applied this methodology to the molecular chaperone Tumor Necrosis Factor Receptor-Associated Protein 1 (TRAP1) and its 11 allosteric modulators, which exhibited a broad spectrum of inhibitory efficacies despite their similar affinities. The trained ML models predicted a significant positive correlation between the percentage of the inhibition-like TRAP1 state and inhibitory efficacies, highlighting the potency of this framework in quantifying allosteric efficacy.

### De novo allosteric modulator design

A conventional de novo approach for designing novel allosteric molecular structures involves assembling potential functional fragments. Reversely, one could decompose available allosteric modulators into different functional fragments, which are then combined to produce modulators with new scaffolds. Based on this concept, Bian et al. [82] collected all metabotropic glutamate receptor 5 (mGluR5) allosteric modulators from ASD and employed retrosynthetic combinatorial analysis to generate a fragment library. This library was subsequently filtered using basic pharmacological rules and molecular docking to pinpoint crucial fragments for binding. The binding site was defined based on a known allosteric site, as determined by the crystal structure of mGluR5 in complex with the negative allosteric modulator mavoglurant [83]. The identified critical fragments were then reassembled into 9,600 ligands and evaluated by second-round docking and structure–activity relationship analysis. As a result, 20 possible modulators with diverse new scaffolds were obtained.

In recent years, ML-based generative models substantially promote the de novo allosteric modulator design process. Such models strive to extract representative features automatically and learn the distribution of the ligand set in the training dataset, allowing the generation of ligands that bear a resemblance to those in the training set yet also display a degree of novelty. To this end, Bian and Xie [84] trained one of the generative models, long short-term memory recurrent neural networks, on half a million drug-like molecules. Next, they constructed a CB2 target-specific model, named t-DeepMGM, by fine-tuning the generative model on a dataset of reported CB2 modulators. The t-DeepMGM model generated a new CB2 allosteric bioactive ligand, XIE9137. Besides ligand-based modulator generators, target-based generators have also been constructed, which generate potential modulators through complementary pocket information. LiGANN [85] is one such generator based on a generative adversarial network (GAN). This model encodes the voxelized pocket shape into ligand shapes, which are then decoded into valid molecular SMILES strings. Vennila and Elango [86] used the LiGANN to design potential pyruvate dehydrogenase kinase 1 modulators based on the defined allosteric site from 5 representative conformation states. The resulting top-ranking compounds exhibiting new scaffolds and high binding affinity were calculated by MD simulations.

## Rational Allosteric Modulator Design

### A multi-strategy for mining the allosteric target potential: A case study of SIRT6

SIRT6, a member of the human sirtuin family (SIRT1 to SIRT7), is a critical epigenetic target implicated in a range of cellular processes, including cancer tumorigenesis, progression, genomic stability, metabolism, and aging [87]. The selective regulation of SIRT6 catalytic activity presents a formidable challenge due to the conserved substrate binding site and high affinity with cofactors. However, designing allosteric modulators external to the orthosteric site offers a viable strategy.

**Fig. 3.**
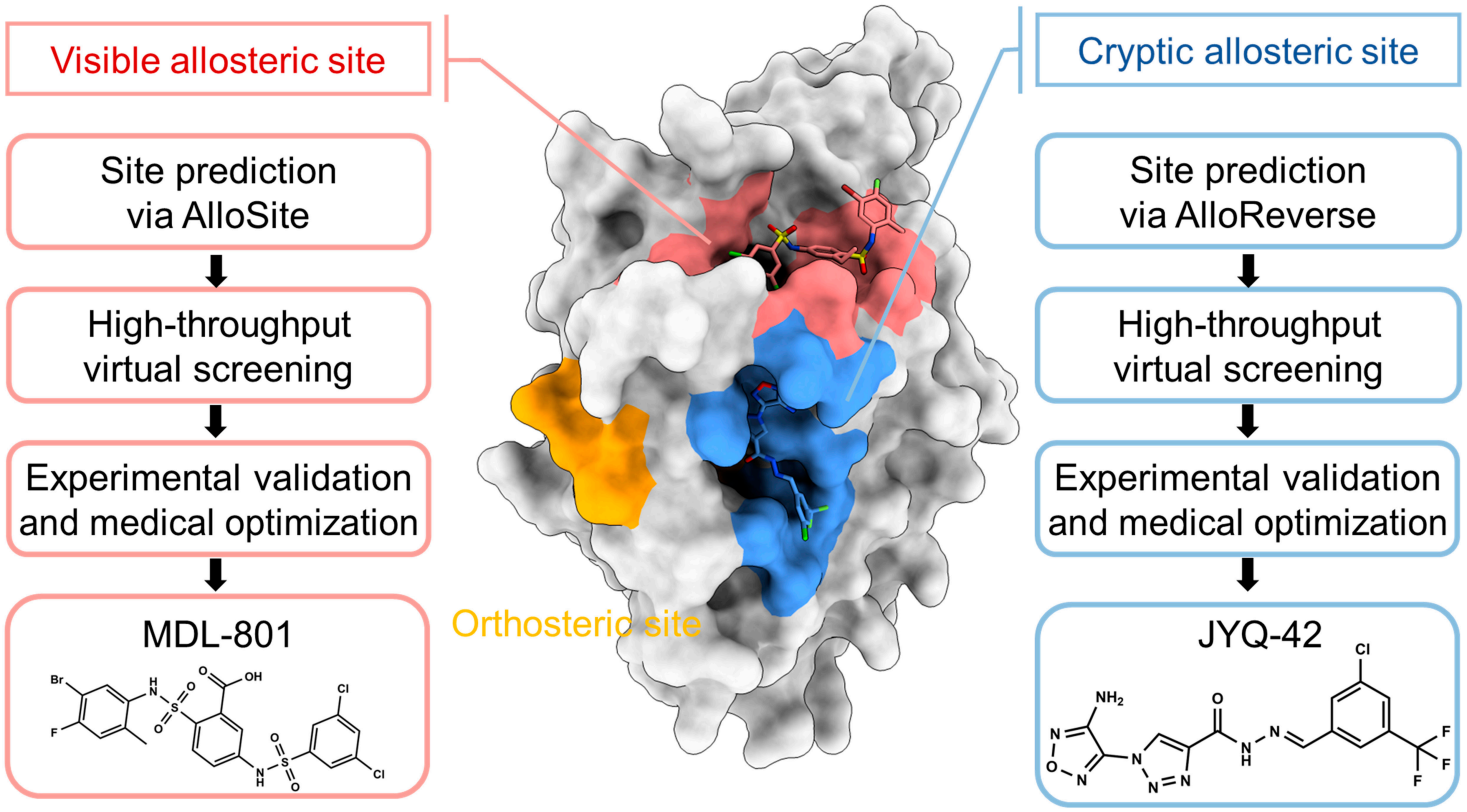
Example of successful rational design of allosteric modulator on SIRT6. The crystal structure of SIRT6 (Protein Data Bank [PDB] code 5Y2F) is presented in a surface representation, with the orthosteric site, visible allosteric site, and cryptic allosteric site distinctly colored orange, red, and blue, respectively. The comprehensive workflows for the discovery of allosteric modulators are graphically illustrated on both sides of the depiction.

Addressing these challenges, our team applied forward and reversed tactics to explore the potential of allosterically targeting SIRT6 (Fig. 3). From the forward perspective [88], we used our AlloSite tool to predict potential visible allosteric sites on the available SIRT6 crystal structure [44]. A deep hydrophobic allosteric site, flanked by residues Phe82 and Phe86, was identified. Based on this potential allosteric site, we virtually docked over 500 million compounds using Glide software. The top 20 compounds displaying high predicted binding affinity and favorable hydrophobicity were selected and procured. Two initial activator hits were identified with deacetylation activity through experimental validation, exhibiting EC_50_ values of 173.8 ± 1.3 μM and 217.6 ± 1.1 μM. Subsequent medicinal chemistry optimizations led to the discovery of 2 activators, MDL-800 and MDL-801, both of which exhibited significantly enhanced EC_50_ values of 10.3 ± 0.3 μM and 5.7 ± 0.3 μM, respectively, and demonstrated high selectivity within the SIRTs family. Importantly, the crystallographic structure and mutagenesis experiments strongly showed that MDL-801 binds to the computationally predicted allosteric site. Moreover, cellular and animal model studies demonstrated that allosteric SIRT6 activators could effectively suppress the proliferation of human hepatocellular carcinoma. This study, thus, facilitates the exploration of allosteric SIRT6 modulators and offers promising therapeutics for associated carcinomas.

Recently, from the reverse perspective [89], our correlation-based AlloReverse platform detected a novel cryptic allosteric site, Pocket Z [70]. This site is highly flexible and transient, only manifesting in the SIRT6 system with orthosteric perturbations. Following virtual screening and optimization, we identified the most potent and selective noncompetitive SIRT6 inhibitor, JYQ42, with an IC_50_ value of 2.33 ± 0.17 μM. Notably, JYQ-42 can significantly diminish SIRT6-mediated cancer cell migration and suppress pro-inflammatory cytokine expression in pancreatic cancer cells. Collectively, this case study presents a generalized multi-strategy approach to demonstrate the allosteric potential of targets.

### An integrated platform for allosteric modulator design: Verification using STAT3

To date, a plethora of computational methods have been proposed for specific allosteric applications, as we reviewed above. However, their diverse theoretical principles and complex hyperparameter settings limit their extensibility. There is a notable absence of a convenient integrated platform for efficiently identifying allosteric sites, acquiring potential allosteric modulators, and comprehensively assessing the biological allosterism of these modulators. Thereupon, we developed an integrated platform called AlloFinder based on our previous allosteric data and approaches [90]. AlloFinder integrates AlloSite to discover potential allosteric sites and utilizes Allolike to filter “allosteric-like” compounds from either an in-house compound library or user uploads. This platform subsequently performs virtual screening of these “allosteric-like” compounds at the predicted site, resulting in potential hit modulators ranked by Alloscore, an accurate allosteric scoring function. AlloFinder also maps the predicted allosteric site and modulators to allosterome for in-depth analysis. This process could reveal the specificity of the site within the human proteome and guide hit optimization based on known allosteric modulators.

**Fig. 4.**
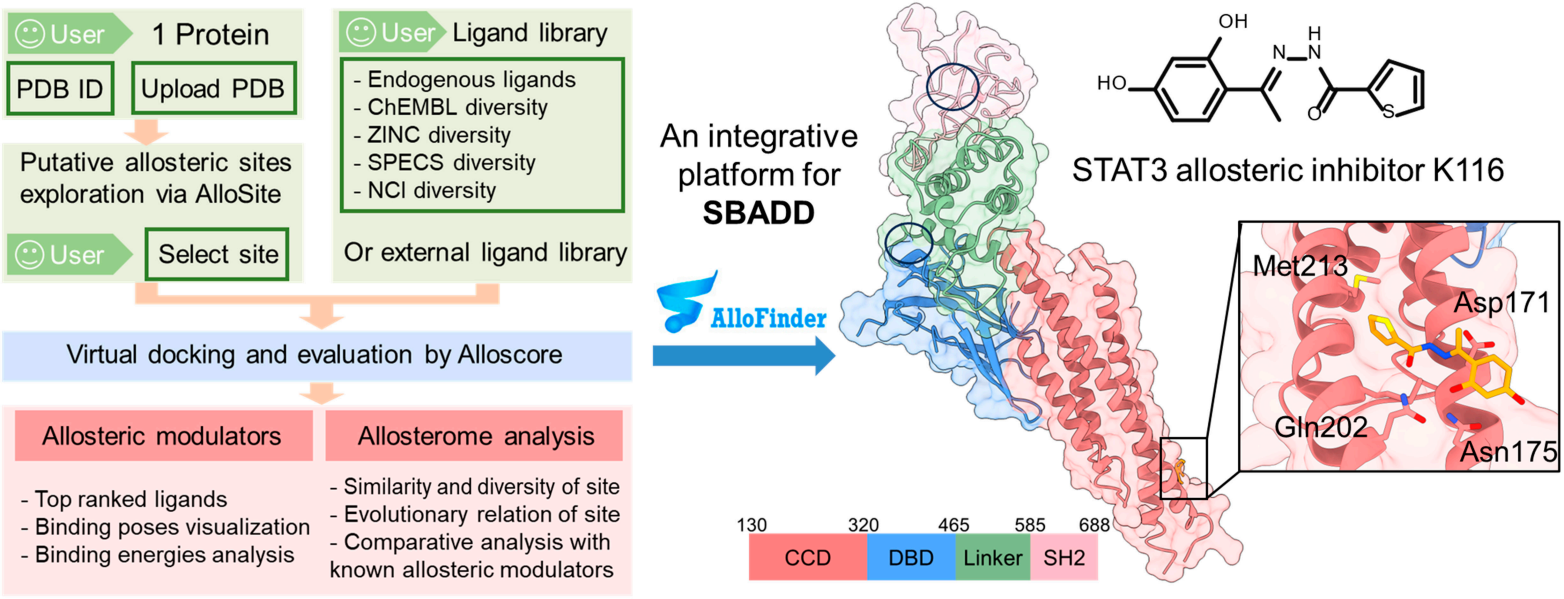
Example of successful rational design of allosteric modulator on STAT3. The crystal structure of STAT3 (PDB code 1BG1) is color-coded according to the domain. The automated AlloFinder tool (the left side shows its overall workflow) facilitates the prediction of the allosteric site at the CCD, virtual screening, and evaluation of allosteric interactions post-docking. An enlarged view exhibits the interactions between the allosteric inhibitor K116 and STAT3, aligning with the findings from mutagenesis experiments. The pockets in the SH2 domain and DBD are labeled by black circles according to the representative STAT3 inhibitors BMA097 and in S3-54, respectively.

Importantly, the ability of AlloFinder has been verified by identifying a novel allosteric modulator targeting the Signal transducer and activator of transcription 3 (STAT3) (Fig. 4) [91]. The aberrant activation of STAT3 is closely linked to various human cancers. Although many inhibitors targeting the Src homology 2 (SH2) domain and DNA-binding domain (DBD) have been developed, these often prove ineffective in clinical trials due to poor pharmacokinetics and high cytotoxicity [92]. Designing allosteric modulators binding to the coiled-coil domain (CCD) may provide a therapeutic solution. AlloFinder identified 5 potential allosteric sites, including one at the CCD, and discovered an allosteric inhibitor with a *K*_d_ value of 3.22 ± 0.85 μM toward the CCD site. Further mutagenesis experiments were confirmed as a bona fide site, and cellular experiments demonstrated that the modulator could inhibit STAT3 and promote STAT3-mediated cellular apoptosis [90].

## Concluding Remarks and Future Perspectives

Allostery has been dubbed the “second secret of life”, second only to the genetic code, due to its ubiquitous role in physiological processes and pathological developments, thus garnering considerable pharmaceutical interest [93]. Many critical therapeutic targets have historically been considered undruggable or challenging to target using traditional orthosteric drug discovery strategies, given their competitive nature, indefinite site, and poor selectivity. However, allosterism offers a valuable approach for regulating these targets, with an increasing number of allosteric therapeutic agents undergoing clinical evaluation, such as Ras GTPase [94], SHP2 kinase [95], and GPCRs [96]. These agents distinguish themselves from orthosteric drugs by demonstrating superior selectivity, resulting in fewer side effects and lower toxicity [8,9]. The advantages of allosteric drug design, coupled with the availability of vast allosteric data and the advent of powerful computational methodologies, have spurred extensive research into developing rational procedures and efficient toolkits specifically for allosteric drug design [15]. Consequently, allosteric drug discovery has evolved from being traditionally serendipitous to becoming a more systematized SBADD approach.

**Fig. 5.**
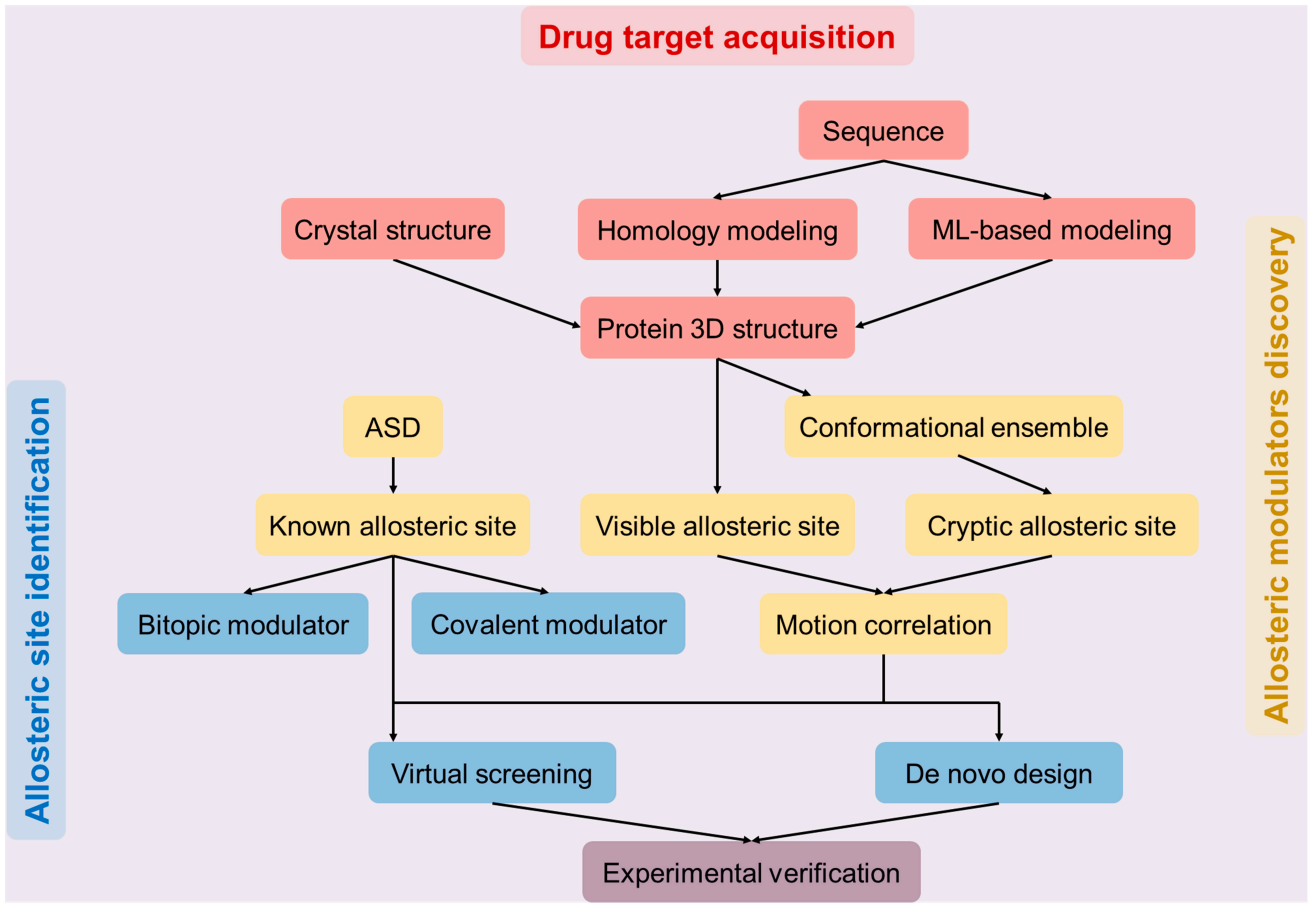
A proposed pipeline for SBADD.

However, the intricate nature of allosteric modulation confounds the extraction of a universal SBADD procedure. The methodologies adopted are largely contingent upon the context (Fig. 5). In the instance where the 3D structure of the interested target remains unresolved, homology modeling or extraction of high-confidence proportions from the AF2-predicted structure presents a viable starting point. Crucially, the reliability of the predicted structure should either be examined via molecular docking to identify known allosteric modulators among inactive decoy sets [97] or be improved via extracting the average conformation from ensembles generated by MD simulations [98]. Once the reliable 3D structure is determined, if the allosteric site is well delineated from literature or experimental data, standard rational drug design procedures—encompassing virtual screening, MD simulations, and medical optimization—may be executed. Otherwise, site detection tools could be employed to detect visible allosteric cavities in the protein structure [44–55]. In cases where allosteric pockets do not visibly emerge or are inadequately defined, cryptic binding sites can be revealed through the exploration of conformational ensembles generated by MD simulation [59–61,64,65]. A wealth of studies suggests that exploring the long-range coupling between these putative allosteric sites and orthosteric sites is of vital importance for determining the allosteric site’s druggability [9,57,99]. In addition, when residues such as cysteine, serine, threonine, or tyrosine surround the potential allosteric site, the design of covalent allosteric modulators capable of irreversibly binding to these residues with increased efficacy may be considered. When the potential allosteric site is in close proximity to known orthosteric or other allosteric sites housing known modulators, bitopic modulators could be conceived. Beyond these cases, conventional virtual screening with classical score functions or a specifically designed Alloscore [79] or de novo allosteric modulator generation can yield high-ranking candidate allosteric modulators for subsequent experimental validation.

Currently, allosteric modulator discovery continues to face multiple challenges. Firstly, the effectiveness of ML-based allosteric site detection models and Alloscore is hampered by previous small datasets. However, the considerable influx of novel allosteric sites and modulator-receptor co-crystals characterized and archived in ASD has broadened the training sets, facilitating the creation of more robust and accurate ML-based predictive models. Secondly, allosteric sites are often treated with the same rigidity as orthosteric sites during docking or virtual screening processes, underestimating the inherent flexibility of allosteric sites and the intricacies of allosteric regulation. Recent advances in computational capabilities could allow for a more comprehensive consideration of receptor flexibility. Thirdly, despite the efficacy of computer-assisted identification of allosteric hit compounds, the hit-to-lead process—wherein medical optimizations transform a hit into a promising lead compound—has been comparatively sluggish due to a scarcity of systematic and reliable data. To address this, we are preparing a high-quality allosteric hit-to-lead dataset, which could serve as a pivotal foundation for the development of corresponding computational algorithms. Finally, while numerous studies on the design of allosteric modulators concentrate primarily on the micro perspective at a single-molecule level, our understanding of the macro perspective of allostery at the cellular level remains relatively limited. By delving into the exploration of allostery within cellular networks, we could broaden the scope for identifying allosteric modulators, potentially leading to the discovery of allo-network drugs.

Nevertheless, the recent SBADD paradigm has achieved notable success in the rational design of allosteric modulators over the past decade. This success has sparked considerable interest from academia and industry, prompting further enhancements. With the steady influx of extensive allosteric data and considerable advancements in computational capabilities and methodologies, particularly the remarkable success of big models, we expect SBADD to evolve toward a more automated and efficient phase. In the not-so-distant future, we anticipate the development of an AlloGPT platform analogous to ChatGPT in the field of allostery. This platform would endow us with ChatGPT-like capabilities for exploring allostery and rationally designing allosteric modulators, propelling the field of allosteric research and drug discovery into new frontiers.
